# Response: Concerns with conclusions in the article by Sherwood *et al* ‘Key differences between 13 KRAS mutation detection technologies and their relevance for clinical practice’

**DOI:** 10.1136/esmoopen-2017-000294

**Published:** 2018-01-03

**Authors:** James Sherwood

**Affiliations:** Innovative Medicines Biotech Unit, Precision Medicine and Genomics, Cambridge, UK

**Keywords:** kras

## Abstract

Click here and here to see the linked papers

To the Editor,

We thank Dr Karlin-Neumann for his comments^1^ on our work^2^ and welcome the opportunity to provide clarification on the points raised.

We carried out a thorough review of the droplet digital PCR (ddPCR) data and the overall study. In summary, we determine that the overall conclusions of the study are valid. The reduced sensitivity and specificity of the ddPCR assay were a result of high background signals inherent with the ddPCR reagents as supplied at the time to the testing laboratory (IMGM, Munich). Notwithstanding this overall conclusion and finding, our investigation did also identify an error at IMGM laboratory (discovered on 29 October 2017), where one of the five assays used was incorrect and codon Q61 data should not have been included. Therefore, we outline corrections to the published work as detailed below.

We are confident that concerns over bias in the study are unfounded as great care was taken to ensure balanced representation of each technology in respect of data, commentary and relevant references. We also conclude that the fact the data do not show superiority of one technology over another and other limitations are clearly stated, and therefore that the concerns raised are not valid.

Please find our detailed analysis and findings in the discussion below.

Concerning the study design, we can confirm that the same amount of sample material was analysed by all of the technologies tested, that is, 50 or 100 mutant copies per test reaction, and that the input DNA sample was diluted according to the volume requirements as described in each technology section of the online supplementary methods. Further, we can confirm—as stated in our publication (page 3)—that one test per sample was permitted unless part of an established repeat testing procedure as outlined in the online supplementary methods.

For the ddPCR method, we would like to clarify that indeed 1×50 and 1×100 mutant copies per reaction were used, diluted into 9 µL DNA in a final reaction volume of 20 µL. We acknowledge that the discrepancy between our results and reported data on the Bio-Rad ddPCR assay and its performance may not be as expected. However, as discussed in the manuscript (page 8), this discrepancy may be caused by the use of cell line admixtures to mimic clinical formalin-fixed paraffin-embedded (FFPE)-derived samples and that local validation of a given assay using this sample type would be necessary.

Moreover, as stated, we did observe non-specific results, particularly in the 50 mutant copy samples. On further investigation of the data, we would attribute the unexpectedly low sensitivity for KRAS codons 12 and 13 to high background signals, which made interpretation of the blinded data difficult.

As pointed out by Dr Karlin-Neumann, a detailed description of the method applied to generate the data using the research-use-only Oncomine Focus Assay (ThermoFisher Scientific, Waltham, MA, USA) is provided in the online supplementary document, that is, stating the preparation of replicate libraries for low allele fraction clinical testing applications. Notably, the protocol that was used in this study at the ThermoFisher Scientific laboratory—acting as an independent clinical laboratory demonstrating real-world capabilities with research-use-only components—was originally developed to support testing for cell free DNA (cfDNA) and implemented as a validated laboratory-developed test, including the preparation of replicate libraries for low allele fraction clinical testing applications.

We acknowledge that the Bio-Rad ddPCR mutation assays have by far the highest sensitivity, that is, 0.001% (table 4 in the publication). Further, we do cite Sacher *et al*^3^ which describes the successful validation of the PrimePCR endothelial growth factor receptor gene (EGFR) and KRAS assays on circulating tumour DNA (ctDNA) against matched tumour specimens. We also cite Pender *et al*^4^ which employs the same PrimePCR assays from Bio-Rad and cites cross-reactivity of the assays with the mutant DNA species present in the assays and the need for optimisation using orthogonal methods. We would therefore consider that we reference publications that are relevant to the work conducted in our study, and that they provide a balance on the performance of the Bio-Rad PrimePCR ddPCR mutation assays in other laboratories.

Yet, we would like to highlight regarding the differences between assay and platforms, the manuscript specifically clarifies on page 8 that this study does not show or claim to show superiority of one technology over another. As outlined, this study was carried out in various clinical laboratories (contract), research organisations and in part by assay manufacturers as documented in Supplemental Methods table 1, using a broad range of established and emerging assays to display the characteristics of each. We feel strongly that the limitations of our study, that is, variability in performance across the testing platforms and assays and the fact that it is not intended to show the superiority of one technology over another, are clearly stated.

We have no concerns that regarding the highlighted examples concerning the data from the Idylla KRAS Mutation Test, Oncomine Focus Assay or UltraSEEK assay were influenced by relying on the vendors themselves to have run and analysed their own systems. In fact, 8 of the 13 technologies were run by laboratories that were not manufacturers (see table 1 in Supplemental Methods). The selection was based on the capabilities and expertise of participating laboratories (AstraZeneca, IMGM, Newgene), availability of emerging tests (eg, Illumina TruSight Tumor 15, Agena UltraSEEK) or in the case of Biocartis Idylla, the need to directly add DNA into the test workflow rather than tissue, which was performed by Biocartis, Mechelen, Belgium, since this particular utility was only available to the manufacturer.

We would like to inform that Dr Karlin-Neumann’s correspondence has led to a thorough investigation regarding the conduct of the ddPCR experiments, which were carried out between July and December 2015. Importantly, the investigations discovered on 29 October 2017 by the participating third-party laboratory (IMGM, Munich) that the ddPCR experiments were performed using the incorrect PrimePCR KRAS Q61H mutation assay. In detail, the p.Q61H c.183A>C reagent had been ordered and used instead of the p.Q61H c.183A>T assay (see table 1 in the manuscript). This explains why the PrimePCR KRAS Q61H assay did not detect Q61H mutations correctly, impacting the following sections in the publication: table 3 and figure 1 legend, and related ddPCR results as listed in the abstract and results sections table 1.

**Table 3:** *KRAS* mutation detection success by codon, concentration and technology

(A) 100 Mutant copies input													
	Nominal total copies of WT DNA	Real-time quantitative PCR			MALDI-TOF		Next-generation sequencing					Droplet digital PCR	Sanger capillary sequencing
		therascreen *KRAS* RGQ PCR Kit	cobas *KRAS* Mutation Test	Idylla *KRAS* Mutation Test (point of care)	iPlex Pro	UltraSEEK	Thunderbolts	Oncomine Focus Assay	Sentosa SQ NSCLC Panel	Ion AmpliSeq Cancer Hotspot panel V.2	TruSight Tumor 15	PrimePCR ddPCR Mutation Assays *KRAS*	ABI3730 sequencing
p.G12C	20	✓ MD	✓ MD	✓ MD	✓ MD	✓ MD	✕ NMD	✓ MD 14.3%	✓ MD 20.5%	✓ MD 11.1%	NMD	✕ IMD	✓ NMD
	10	✓ MD	✓ MD	✓ MD	✓ MD	✓ MD	✓ MD 4.7%	✓ MD 6.6%	✓ MD 5.7%	✓ MD 5.7%	✓ MD 4.8%	✓ MD	✓ NMD
	5	✓ MD	✓ NMD	✓ MD	✓ MD	✓ MD	✓ MD 3.7%	✓ MD 2.4%	✓ NMD 3.1%	✓ MD 2.6%	✓ MD 2.7%	✓ MD	✓ NMD
	1	✓ NMD	✓ NMD	✓ MD	✓ MD	✓ MD	✓ NMD	✓ MD 0.5%	✓ NMD –	✓ NMD 0.0%	✓ NMD 0.0%	✓ MD	✓ NMD
	0.5	✓ NMD	✓ NMD	✓ MD	✓ NMD	✓ MD	✓ NMD	✓ MD 0.3%	✓ NMD –	✓ NMD 0.0%	✓ NMD 0.0%	✕ NMD	✓ NMD
p.G12D	20	✓ MD	✓ MD	✓ MD	✓ MD	✓ MD	✓ MD 17.2%	✓ MD 19.6%	✓ MD 25.6%	✓ MD 27.5%	✓ MD 31.1%	✓ MD	✓ NMD
	10	✓ MD	✓ MD	✓ MD	✓ MD	✓ MD	✓ MD 11.4%	✓ MD 11.8%	✓ MD 14.3%	✓ MD 13.2%	✕ NMD	✓ MD	✓ NMD
	5	✓ MD	✓ MD	✓ MD	✓ MD	✓ MD	✓ MD 7.6%	✓ MD 5.8%	✓ MD 6.7%	✓ MD 5.1%	✓ MD 7.3%	✓ MD	✓ NMD
	1	✓ NMD	✓ NMD	✓ MD	✓ NMD	✓ MD	✓ MD 1.5%	✓ MD 1.2%	✓ NMD –	✓ MD 1.0%	✓ MD 1.6%	✓ MD	✓ NMD
	0.5	✓ NMD	✓ NMD	✓ MD	✓ NMD	✓ MD	✓ NMD	✓ MD 0.6 %	✓ NMD –	✓ NMD 0.0 %	✓ NMD 0.0 %	✕ NMD	✓ NMD
p.G13D	20	✓ MD	✓ MD	✓ MD	✓ MD	✓ MD	✕ NMD	✓ MD 13.9%	✓ MD 14.6%	✓ MD 10.4%	✕ NMD	✓ MD	✓ NMD
	10	✓ MD	✓ MD	✓ MD	✓ MD	✓ MD	✓ MD 2.7%	✓ MD 7.4%	✓ MD 7.7%	✓ MD 8.6%	✓ MD 6.4%	✓ MD	✓ NMD
	5	✓ NMD	✓ MD	✓ MD	✓ MD	✓ MD	✓ MD 3.9%	✓ MD 3.1%	✓ MD 4.6%	✓ MD 1.6%	✓ MD 3.8%	✓ MD	✓ NMD
	1	✓ NMD	✓ NMD	✓ MD	✓ NMD	✓ MD	✓ NMD	✓ MD 0.9%	✓ NMD –	✓ NMD 0.0%	✓ NMD 0.0%	✓ MD	✓ NMD
	0.5	✓ NMD	✓ NMD	✓ MD	✓ NMD	✓ MD	✕ NMD	✓ MD 0.4%	✓ NMD –	✓ NMD 0.0%	✓ NMD 0.0%	✕ NMD	✓ NMD
p.G12V	20	✓ MD	✓ MD	✓ MD	✓ MD	✓ MD	✕ NMD	✓ MD 30.6%	✓ MD 39.4%	✓ MD 27.8%	NMD	✓ MD	✓ NMD
	10	✓ MD	✓ MD	✓ MD	✓ MD	✓ MD	✓ MD 5.9%	✓ MD 16.2%	✓ MD 24.5%	✓ MD 15.6%	✓ MD 13.6%	✕ IMD	✓ NMD
	5	✓ MD	✓ MD	✓ MD	✓ MD	✓ MD	✓ MD 6.1%	✓ MD 8.1%	✓ MD 9.6%	✓ MD 8.3%	✓ MD 8.5%	✓ MD	✓ NMD
	1	✓ MD	✓ MD	✓ MD	✓ MD	✓ MD	✓ NMD	✓ MD 1.3%	✓ NMD 1.6%	✓ MD 1.4%	✓ MD 1.5%	✓ MD	✓ NMD
	0.5	✓ MD	✓ NMD	✓ MD	✓ NMD	✓ MD	✓ NMD	✓ MD 1.0%	✓ NMD –	✓ NMD 0.0%	✓ NMD 0.0%	✕ NMD	✓ NMD
p.Q61H	20	NA	✓ MD	✓ MD	✓ MD	✓ MD	✕ NMD	✓ MD 6.3%	✓ MD 7.8%	✓ MD 1.1%	✕ NMD	NP	✓ NMD
	10	NA	✓ NMD	✓ MD	✓ NMD	✓ MD	✓ MD 2.3%	✓ MD 2.7%	✓ NMD 2.4%	✓ MD 3.0%	✓ MD 4.3%	NP	✓ NMD
	5	NA	✓ NMD	✓ MD	✓ NMD	✓ MD	✓ NMD	✓ MD 1.2%	✓ NMD 2.5%	✓ MD 1.8%	✓ MD 1.5%	NP	✓ NMD
	1	NA	✓ NMD	✓ MD	✓ NMD	✓ NMD	✓ NMD	✓ MD 0.2%	✓ NMD –	✓ NMD 0.0%	✓ NMD 0.0%	NP	✓ NMD
	0.5	NA	✓ NMD	✓ MD	✓ NMD	✓ NMD	✓ NMD	✓ MD 0.1%	✓ NMD –	✓ NMD 0.0%	✓ NMD 0.0%	NP	✓ NMD

(B) 50 Mutant copies input													
	Nominal total copies of WT DNA	Real-time quantitative PCR			MALDI-TOF		Next-generation sequencing					Droplet digital PCR	Sanger capillary sequencing
		therascreen *KRAS* RGQ PCR Kit	cobas *KRAS* Mutation Test	Idylla *KRAS* Mutation Test (point of care)	iPlex Pro	UltraSEEK	Thunderbolts	Oncomine Focus Assay	Sentosa SQ NSCLC Panel	Ion AmpliSeq Cancer Hotspot panel V.2	TruSight Tumor 15	PrimePCR ddPCR Mutation Assays *KRAS*	ABI3730 sequencing
p.G12C	20	✕ NMD	✓ MD	✓ MD	✓ MD	✓ MD	✕ NMD	✓ MD 10.8%	✓ MD 19.8%	✓ MD 13.1%	✕ NMD	✓ MD	✓ NMD
	10	✓ MD	✓ MD	✓ MD	✓ NMD	✓ MD	✕ NMD	✓ MD 5.8%	✓ MD 6.3%	✓ MD 7.1%	✕ NMD	✓ MD	✓ NMD
	5	✓ NMD	✓ NMD	✓ MD	✓ NMD	✓ MD	✓ MD 2.1%	✓ MD 2.9%	✓ MD 3.3%	✓ MD 1.9%	✓ MD 3.6%	✓ MD	✓ NMD
	1	✓ MD	✓ NMD	✓ MD	✓ NMD	✓ MD	✓ NMD	✓ MD 0.6%	✓ NMD –	✓ NMD 0.0%	✓ NMD 0.0%	✓ MD	✓ NMD
	0.5	✓ NMD	✓ NMD	✓ MD	✓ NMD	✓ MD	✓ NMD	✓ MD 0.4%	✓ NMD –	✓ NMD 0.0%	✓ NMD 0.0%	✕ IMD	✓ NMD
p.G12D	20	✕ NMD	✓ MD	✓ MD	✓ MD	✓ MD	✕ NMD	✓ MD 22.0%	✓ MD 16.6%	✓ MD 23.2%	✕ NMD	✓ MD	✓ NMD
	10	✓ MD	✓ MD	✓ MD	✓ MD	✓ MD	✓ MD 9.3%	✓ MD 11.5%	✓ MD 18.4%	✓ MD 8.4%	✓ MD 9.7%	✓ MD	✓ NMD
	5	✓ MD	✓ MD	✓ MD	✓ MD	✓ MD	✓ MD 5.0%	✓ MD 5.0%	✓ MD 5.2%	✓ MD 7.6%	✓ MD 6.7%	✓ MD	✓ NMD
	1	✓ NMD	✓ NMD	✓ MD	✓ MD	✓ MD	✓ NMD	✓ MD 1.2%	✓ NMD 2.2%	✓ MD 1.9%	✓ NMD 0.0%	✓ MD	✓ NMD
	0.5	✓ NMD	✓ NMD	✓ MD	✓ NMD	✓ MD	✓ NMD	✓ MD 0.3%	✓ NMD –	✓ MD 1.2%	✓ NMD 0.0%	✕ IMD	✓ NMD
p.G13D	20	NMD	✓ MD	✓ MD	✓ MD	✓ MD	✕ NMD	✓ MD 13.0%	✓ MD 6.5%	✓ MD 15.5%	✕ NMD	✓ MD	✓ NMD
	10	✓ MD	✓ MD	✓ MD	✓ MD	✓ MD	✕ NMD	✓ MD 6.6%	✓ MD 6.6%	✓ MD 6.1%	✓ MD 2.7%	✕ IMD	✓ NMD
	5	✓ MD	✓ MD	✓ MD	✓ MD	✓ MD	✓ MD 2.8%	✓ MD 2.9%	✓ NMD 2.5%	✓ MD 2.0%	✓ MD 2.6%	✓ MD	✓ NMD
	1	✓ NMD	✓ NMD	✓ MD	✓ NMD	✓ MD	✓ NMD	✓ MD 0.8%	✓ NMD –	✓ NMD 0.0%	✓ NMD 0.0%	✓ MD	✓ NMD
	0.5	✓ NMD	✓ NMD	✓ MD	✓ NMD	✓ MD	✓ NMD	✓ MD 0.4%	✓ NMD –	✓ NMD 0.0%	✓ NMD 0.0%	✕ IMD	✓ NMD
p.G12V	20	✕ NMD	✓ MD	✓ MD	✓ MD	✓ MD	✕ NMD	✓ MD 34.3%	✓ MD 46.9%	✓ MD 29.6%	✓ MD 26.1%	✓ MD	✓ NMD
	10	✓ MD	✓ MD	✓ MD	✓ MD	✓ MD	✕ NMD	✓ MD 17.3%	✓ MD 13.9%	✓ MD 15.3%	✕ NMD	✕ IMD	✓ NMD
	5	✓ MD	✓ MD	✓ MD	✓ MD	✓ MD	✓ MD 5.1%	✓ MD 8.5%	✓ MD 12.2%	✓ MD 7.0%	✓ MD 10.8%	✕ IMD	✓ NMD
	1	✓ MD	✓ NMD	✓ MD	✓ MD	✓ MD	✓ NMD	✓ MD 1.7%	✓ NMD –	✓ MD 1.8%	✓ MD 1.8%	✕ IMD	✓ NMD
	0.5	✓ MD	✓ NMD	✓ MD	✓ NMD	✓ MD	✓ NMD	✓ MD 0.8%	✓ NMD –	✓ NMD 0.0%	✓ NMD 0.0%	✓ MD	✓ NMD
p.Q61H	20	NA	✓ MD	✓ NMD	✓ MD	✓ MD	✕ NMD	✓ MD 6.0%	✓ MD 10.3%	✓ MD 4.5%	✕ NMD	NP	✓ NMD
	10	NA	✓ MD	✓ MD	✓ MD	✓ MD	✕ NMD	✓ MD 2.0%	✓ NMD 2.8%	✓ MD 3.7%	✕ NMD	NP	✓ NMD
	5	NA	✓ NMD	✓ MD	✓ NMD	✓ MD	✓ NMD	✓ MD 1.7%	✓ NMD –	✓ MD 1.6%	✓ NMD 0.0%	NP	✓ NMD
	1	NA	✓ NMD	✓ NMD	✓ NMD	✓ NMD	✓ NMD	✓ MD 0.4%	✓ NMD –	✓ NMD 0.0%	✓ NMD 0.0%	NP	✓ NMD
	0.5	NA	✓ NMD	✓ MD	✓ NMD	✓ NMD	✓ NMD	✓ MD 0.3%	✓ NMD –	✓ NMD 0.0%	✓ NMD 0.0%	NP	✓ NMD

(C) Wild-type only												
No of copies	Real-time quantitative PCR			MALDI-TOF		Next-generation sequencing					Droplet digital PCR	Sanger capillary sequencing
	therascreen *KRAS* RGQ PCR Kit	cobas *KRAS* Mutation Test	Idylla *KRAS* Mutation Test (point of care)	iPlex Pro	UltraSEEK	Thunderbolts	Oncomine Focus Assay	Sentosa SQ NSCLC Panel	Ion AmpliSeq Cancer Hotspot panel V.2	TruSight Tumor 15	PrimePCR ddPCR Mutation Assays *KRAS*	ABI3730 sequencing
20 000	✓ WT	✓ WT	✓ WT	✓ WT	✓ WT	✓ WT	✓ WT	✓ WT	✓ WT	✕ NMD	✓ NMD	✓ WT
10 000	✓ WT	✓ WT	✓ WT	✓ WT	✓ WT	✓ WT	✓ WT	✓ WT	✓ WT	✓ WT 0.0%	✕ NMD	✓ WT
2000	✓ WT	✓ WT	✓ WT	✓ WT	✓ WT	✓ WT	✓ WT	✓ WT	✓ WT	✓ WT 0.0%	✕ NMD	✓ WT
1000	✓ WT	✓ WT	✓ WT	✓ WT	✓ WT	✓ WT	✓ WT	✓ WT	✓ WT	✕ NMD	✓ NMD	✓ WT
500	✓ WT	✓ WT	✓ WT	✓ WT	✓ WT	✕ NMD	✓ WT	✓ WT	✓ WT	✕ NMD	✕ NMD	✓ WT
250	✓ WT	✓ WT	✓ WT	✓ WT	✓ WT	✕ NMD	✓ WT	✓ WT	✓ WT	✕ NMD	✕ NMD	✓ WT

✓ MD, analysis successful, mutation detected; ✓ NMD, analysis successful, but no mutation detected (in the case of the Sentosa assay, a mutation was detected but deemed to be below the defined cut-off); ✓ WT, analysis successful, wild-type sample; ✕ NMD, analysis unsuccessful, no mutation detected; ✕ IMD, incorrect mutation detected; NA, kit does not assay codon; NP, not performed.

MALDI-TOF, matrix-assisted laser desorption/ionisation time-of-flight mass spectrometry.

Consequently, a revised table 3 is given in the online supplementary file, Appendix 1. In the revised table 3A (100 mutant copies input) and 3B (50 mutant copies input), data for the p.Q61H mutation now depict ‘not performed; NP’ as explained in a revised footnote; there are no changes to table 3C.

With respect to other changes that are required to the published report to correct for the mistake on codon 61 ddPCR data, we can clarify:

1. For figure 1 legend: The text should explain that for the ddPCR assay, only four and not five assays were assessed, explaining that a correction is required due to the discovery of an error where the investigating laboratory used the incorrect Q61H assay for the ddPCR technology. ‘Note: The therascreen KRAS RGQ PCR Kit Q61 assay does not test for Q61H; the PrimePCR ddPCR Mutation Assay was not performed for Q61H due to an error at the participating laboratory’.
2. With respect to the calculations in the abstract, the corrected sentence should now reflect a lower number of total data points (718 instead of 728) because of the removal of 5 data points for ddPCR p.Q61H mutation results for 100 mutant copies input and 5 data points for 50 mutant copies input, respectively: ‘Overall 406/718 data points across all 13 technologies were identified correctly’. And ‘The digital PCR assay (KRAS PrimePCR ddPCR, Bio-Rad Laboratories) identified 70% (100 copies) and 65% (50 copies) of samples correctly’.
3. Lastly, the ddPCR results section is corrected to reflect that for codon Q61H, the incorrect PrimePCR KRAS mutation assay had been used (p.Q61H c.183A>C instead of p.Q61H c.183A>T assay): ‘The PrimePCR ddPCR KRAS Mutation Assays were able to identify codon 12 and 13 mutations down to 1% with the 100 copy input. However, across both admixture and wild-type control samples the assay identified the incorrect mutation in nine different mutation/allele frequency combinations (see table 3). When performing the p.Q61H assay, a mistake was made and the incorrect reagent, i.e. detection of c.183A>C instead of c.183A>T, was used. Therefore, table 3 and figure 1 reflect data only for codons 12 and 13’.

We further agree that ddPCR would have offered an absolute quantitation result, and indeed we were planning to include this when the study was designed. However, before unblinding the plate layout and sample annotation, the participating third-party laboratory (IMGM, Munich) had observed that mutant allele frequencies (MAF%) were not consistently available for all samples, mainly due to increased background signals in the codon G12/G13 assays. Moreover, mutant allele frequencies in some of these samples had shown signals for more than one of the *KRAS* mutations known to be present in the materials. Therefore, it did not appear to be appropriate to represent the MAF% and it was considered of interest to display all the next-generation sequencing (NGS) technology mutant allele frequencies instead. Of note, when the ddPCR results were reanalysed after unblinding in an exploratory analysis, a high concordance with MAF% as detected by NGS assays was observed. However, as this was performed after unblinding of the identity of the samples, the experimental set-up did not allow inclusion of these data in the publication.

Further, we can confirm that appropriate ‘no template controls’ in quadruplicates were run to assess possible contamination. The participating third-party laboratory (IMGM, Munich) also confirmed that for each wild-type and *KRAS* mutant assay pair, a wild-type (negative) control was included in duplicates, with thresholds of droplet clusters set manually. The fact that no samples containing fixed percentages of mutant DNA copies were available for analysis did not affect the ability to manually set the threshold to the background signal.

We appreciate that a number of publications underline the sensitivity of ddPCR technology, as outlined in the particular review mentioned by Olmedillas-López *et al*.^5^ However, we do not believe that our results contradict the work that these laboratories and publications describe with our data. For example, we read with interest the publication by Oxnard *et al*^6^ where excellent performance was demonstrated on clinical samples when using custom-made primer/probe mix reagents by Life Technologies for targets such as EGFR T790M, EGFR L858R, EGFR exon deletion 19, BRAF V600E and KRAS G12C (Oxnard *et al* Supplementary methods 1, page 2),^6^ and primer and probe sets benefited from the additional specificity and additional sensitivity that Life Technologies minor groove binder probes offer. Concerning Whale *et al*^7^ we understand that in their publication, four samples were used, which were manufactured from plasmid fragments made from synthetic cloned and purified DNA, which in our experience are preferentially amplifiable clean templates. As such, we would assume that there are also limitations to these models as they tend to be much easier to detect than corresponding amounts of non-synthetic DNA and do not typically perform in the same way as genomic DNA template. Therefore, we do not consider that the performance of the assays highlighted in these studies are relevant to the performance you would expect on the cell line models used using the PrimePCR ddPCR Mutation Assays.

In summary, our study^2^ was carried out in various clinical laboratories (contract), research organisations and in part by assay manufacturers highlighting to laboratories the need for robust local validation. Further, the limitations of the study and the fact that it does not show the superiority of one technology over another are clearly stated and thereby offer a valuable resource regarding the issues faced by diagnostic laboratories.

We acknowledge the discrepancy between our results using contrived admixture cell line samples with challenging low levels of DNA and reported data on the Bio-Rad ddPCR technology, and that the performance may not be as expected by the manufacturer. Data on the incorrect use of c.183A>C instead of c.183A>T to assess mutations for p.Q61H are suggested to be corrected to explain to the readers that the participating third-party laboratory (IMGM, Munich) made a mistake during study conduct for this particular codon. However, we do not consider that this would change the overall conclusion of the study.

We hope our explanations address the questions and concerns^1^ raised by Dr Karlin-Neumann.

## References

[1] Karlin-Neumann G Concerns with conclusions in the article by Sherwood et al ‘Key differences between 13 KRAS mutation detection technologies and their relevance for clinical practice’. ESMO Open. In Press 2017. 10.1136/esmoopen-2017-000287 PMC5757476 29345689

[2] Sherwood JL, Brown H, Rettino A, et al Key differences between 13 KRAS mutation detection technologies and their relevance for clinical practice. ESMO Open 2017;2:e000235 10.1136/esmoopen-2017-000235 29018576 PMC5623342

[3] Sacher AG, Paweletz C, Dahlberg SE, et al Prospective validation of rapid plasma genotyping for the detection of EGFR and KRAS mutations in advanced lung cancer. JAMA Oncol 2016;2:1014–22. 10.1001/jamaoncol.2016.0173 27055085 PMC4982795

[4] Pender A, Garcia-Murillas I, Rana S, et al Efficient genotyping of kras mutant non-small cell lung cancer using a multiplexed droplet digital PCR approach. PLoS One 2015;10:e0139074 10.1371/journal.pone.0139074 26413866 PMC4586384

[5] Olmedillas-López S, García-Arranz M, García-Olmo D Current and emerging applications of droplet digital PCR in oncology. Mol Diagn Ther 2017;21:493–510. 10.1007/s40291-017-0278-8 28477149

[6] Oxnard GR, Paweletz CP, Kuang Y, et al Noninvasive detection of response and resistance in EGFR-mutant lung cancer using quantitative next-generation genotyping of cell-free plasma DNA. Clin Cancer Res 2014;20:1698–705. 10.1158/1078-0432.CCR-13-2482 24429876 PMC3959249

[7] Whale AS, Devonshire AS, Karlin-Neumann G, et al International interlaboratory digital PCR study demonstrating high reproducibility for the measurement of a rare sequence variant. Anal Chem 2017;89:1724–33. 10.1021/acs.analchem.6b03980 27935690

